# National surveillance of *Salmonella enterica *in food-producing animals in Japan

**DOI:** 10.1186/1751-0147-51-35

**Published:** 2009-08-25

**Authors:** Kanako Ishihara, Toshio Takahashi, Ayako Morioka, Akemi Kojima, Mayumi Kijima, Tetsuo Asai, Yutaka Tamura

**Affiliations:** 1 National Veterinary Assay Laboratory, Ministry of Agriculture, Forestry and Fisheries, 1-15-1 Tokura, Kokubunji, Tokyo 185-8511, Japan; 2 Department of Veterinary Public Health, School of Veterinary Medicine, Rakuno Gakuen University, Ebetsu, Hokkaido 069-8501, Japan

## Abstract

A total of 518 fecal samples collected from 183 apparently healthy cattle, 180 pigs and 155 broilers throughout Japan in 1999 were examined to determine the prevalence and antimicrobial susceptibility of *Salmonella*. The isolation rates were 36.1% in broilers, 2.8% in pigs and 0.5% in cattle. *S. enterica *Infantis was the most frequent isolate, found in 22.6% of broiler fecal samples. Higher resistance rates were observed against oxytetracycline (82.0%), dihydrostreptomycin (77.9%), kanamycin (41.0%) and trimethoprim (35.2%). Resistance rates to ampicillin, ceftiofur, bicozamycin, chloramphenicol and nalidixic acid were <10%. CTX-M-2 β-lactamase producing *S. enterica *Senftenberg was found in the isolates obtained from one broiler fecal sample. This is the first report of cephalosporin-resistant *Salmonella *directly isolated from food animal in Japan.

## Findings

*Salmonella enterica *is a causative agent of foodborne diseases in humans. In Japan, the Food Poisoning Statistics showed that bacterial food poisoning patients numbered at 10.331 in 2008. Of the patients, salmonellosis is a leading cause accounting for 24.7% (2,551 patients). *S. enterica *Typhimurium was the commonest reported serovar isolated from human cases before 1988. After 1989, *S. enterica *Enteritidis became the predominant serovar, accounting for almost 50% of salmonellosis in humans . As infections with *S. enterica *Enteritidis are closely linked with egg consumption in Japan, the Enforcement Regulations of the Food Sanitation Law (Law No. 23 of 1948) were amended for safe distribution of raw eggs and liquid egg products in 1998. On the remaining cases, animal meats and products are sources of human infections. In Japan, retail meats are contaminated with *Salmonella *at relatively high level in broiler meats, and low levels in pork and beef [1]. *Salmonella *is sometimes isolated from apparently healthy food-producing animals. The subclinical *Salmonella *infected animals can act as a contamination source for meats and products.

The Japanese Veterinary Antimicrobial Resistance Monitoring System (JVARM) was formed in 1999 in response to international concern about antimicrobial resistance [2]. In 1999, JVARM preliminarily investigated the antimicrobial susceptibility of *Escherichia coli *[3], enterococci [4], *Campylobacter *[5], and *Salmonella *in apparently healthy cattle, pigs and broilers on farms to establish entirely microbiological procedure for the national monitoring system. In the present study, we investigated the prevalence of *Salmonella *in apparently healthy food-producing animals, and demonstrated the presence of cephalosporin-resistant isolate of *S. enterica *Senftenberg in a broiler.

A total of 518 fecal samples were randomly collected from apparently healthy cattle (183 samples), pigs (180 samples) and broilers (155 samples) from all 47 prefectures of Japan from June to December 1999, as described previously [3-5]. In brief, four samples per animal species were collected from different farms in each prefecture. Each sample was collected from individual animals. The fecal samples were transported on ice in sterile plastic specimen tubes to our laboratory, and *Salmonella *was isolated within 3 days. One gram of each fecal sample was inoculated into 10 ml of Hajna tetrathionate broth (Eiken Chemical Co., Ltd., Japan), followed by incubation at 42°C for 18 h for first enrichment cultures, and an additional 5–7 days at room temperature for delayed secondary enrichment culture (DSEC). After incubation, each culture was streaked onto desoxycholate-hydrogen sulfate-lactose agar (Eiken Chemical Co., Ltd.) and brilliant green agar (Eiken Chemical Co., Ltd.) plates, each containing 20 μg/ml novobiocin (Wako Pure Chemical Industries, Ltd., Japan) and incubated at 37°C for 18 h. Candidate colonies were identified biochemically by triple sugar iron (TSI) agar (Eiken Chemical Co., Ltd.) and lysine indole motility (LIM) semisolid agar (Eiken Chemical Co., Ltd.). Identification of serovars was performed by slide and tube agglutination tests (Denka Seiken Co., Ltd., Japan), according to the Kauffmann-White scheme. For individual samples, two isolates were selected for the purpose of determining susceptibility. All of the isolates were stored in 10% skim milk at -80°C until use.

The minimum inhibitory concentration (MIC) of 122 *Salmonella *isolates was determined using a standardized agar dilution method, as described by the Japanese Society of Chemotherapy [6], using Mueller-Hinton agar (Becton, Dickinson and Company, USA). The following 15 antimicrobial agents, approved in Japan as veterinary medicines, were tested: ampicillin, ceftiofur, apramycin, dihydrostreptomycin, kanamycin, gentamicin, oxytetracycline, bicozamycin, chloramphenicol, colistin, nalidixic acid, enrofloxacin, ofloxacin, trimethoprim and sulfadimethoxine. *E. coli *NIHJ and *Staphylococcus aureus *209P were used for quality control. MIC resistant breakpoints were defined microbiologically when the MIC distribution of antimicrobials was bimodal.

Detection of the β-lactamase gene was carried out by polymerase chain reaction (PCR), as previously described by Kojima et al. [7]. Nucleotide sequences of both strands were determined, directly on PCR products. The DNA alignments and deduced amino acid sequences were examined using the BLAST program (National Center for Biotechnology Information, USA).

Statistical analysis was performed using the Chi-square test or Fisher's Exact test.

*Salmonella *was isolated from 56 (36.1%, 95% Confidential Intervals [CI_95%_] 28.6–44.2%) of 155 broiler fecal samples, 5 (2.8%, CI_95% _0.9–6.4%) of 180 porcine fecal samples and 1 (0.5%, CI_95%_0–3.0%) of 183 bovine fecal samples. Isolation rates of *Salmonella *were significantly higher in broiler samples than porcine and bovine samples (P < 0.01). Although *Salmonella *was isolated from 39 samples by first enrichment, it was newly isolated from 23 samples by DSEC. Isolation rate of *Salmonella *increased from 7.7% by first enrichment to 12.0% by DSEC. In Okinawa, which is located in southern Japan, the isolation rates of *Salmonella *from rectal swab samples were 18.0% in 688 broiler chickens and 0% in 100 pigs between 1995 and 2004[8]. Recently, two large-scale surveillance studies of *Salmonella *infections in pigs were reported in Japan [9,10]. Kishima et al. [10] demonstrated that the prevalence of fecal carriage of *Salmonella *was 3.1% in 5393 pigs in 2003–2005. Futagawa-Saito et al. [9] showed that *Salmonella *prevalence was 2.2% and 3.3% in pig fecal samples in 1998–1999 (2980 pigs) and 2004–2005 (3791 pigs), respectively. The present results demonstrated similar *Salmonella *isolation rates to those previously reported in Japan. It is difficult to compare the results in other countries because there are variations in sampling methods and methods for isolation of *Salmonella*. Especially, the prevalence of *Salmonella *in the present study may be underestimated because one gram feces were used for the isolation. However, common procedure of *Salmonella *isolation from all animal species studied was used in the current study. As high *Salmonella *isolation rates were found in broiler chickens, further study should be first performed to clarify the actual status of *Salmonella *colonization in broiler chickens.

*Salmonella *isolates were classified into 14 serovars, including 12 serovars in broiler isolates, 2 serovars in porcine isolates and one serovar in bovine isolate (Table 1). *S. enterica *Infantis was the commonest serovar among broilers in this study, as well as in previous studies [8,11]. In Great Britain, serovars Ohio (22.0%), Kedougou (17.1%), Livingstone (12.2%) and Senftenberg (12.2%) were predominant in the isolates of broiler origin between 2005 and 2006 [12]. In Korea, serovars Enteritidis (21.9%), Typhimurium (23.4%) and Tennessee (20.3%) were frequently isolated from broilers in 2002–2003 [13]. Thus the predominant serovar of *Salmonella *found in broilers varies between regions. In the Netherlands, the predominant serovar changed from Typhimurium in 1984–1989 to Enteritidis in 1996–2001 [14]. In Japan, *S. enterica *Infantis was likely to be the predominant serovar among broilers around 1997 [15]. *S. enterica *Infantis, with similar pulsed field gel electrophoresis profiles and resistance patterns, has been prevalent in Japanese broiler flocks for some time [16].

**Table 1:** *Salmonella *serotype distributions by animal origin

Origin of the samples	Serotype	No. of *Salmonella*-positive samples (%)					
(No. of samples)		FE ^a^		DSEC ^b^		total	
Broilers (n = 155)	Infantis	19	(12.3)	16^†^	(10.3)	35	(22.6)^†^
	Hadar	4	(2.6)	0	(0)	4	(2.6)
	Haifa	1	(0.6)	1	(0.6)	2	(1.3)
	Montevideo	2	(1.3)	0	(0)	2	(1.3)
	Schwarzengrund	2	(1.3)	0	(0)	2	(1.3)
	Thompson	2	(1.3)	0	(0)	2	(1.3)
	Augustenborg	1	(0.6)	0	(0)	1	(0.6)
	Blockley	0	(0)	1	(0.6)	1	(0.6)
	Istanbul	0	(0)	1	(0.6)	1	(0.6)
	Newport	1^†^	(0.6)	0	(0)	1	(0.6)^†^
	Schleissheim	1	(0.6)	0	(0)	1	(0.6)
	Senftenberg	1	(0.6)	0	(0)	1	(0.6)
	untypeable	2^†^	(1.3)	3^†^	(1.9)	5	(3.2)^†^
	All serotypes	35	(22.5)	21	(13.5)	56	(36.1)

Pigs (n = 180)	Typhimurium	2	(1.1)	0	(0)	2	(1.1)
	Ohio	0	(0)	1	(0.6)	1	(0.6)
	untypeable	1	(0.6)	1	(0.6)	2	(1.1)
	All serotypes	3	(1.7)	2	(1.1)	5	(2.8)

Cattle (n = 183)	Blockley	1	(0.5)	0	(0)	1	(0.5)

Total		39	(7.5)	23	(4.4)	62	(12.0)

^a ^FE: First enrichment culture

^b^DSEC: delayed secondary enrichment culture

† Among samples, 2 harbored 2 different serotypes (Infantis and untypeable; Newport and untypeable).

In several countries, *S. enterica *Typhimurium is the leading serovar in the isolates from pigs [17]. Futagawa-Saito et al. [9] showed that the predominant serovars were Agona (28.4%) and Typhimurium (17.9%) in 1998–1999 and Typhimurium (32.5%) and Anatum (17.9%) in 2004–2005. Kishima et al. [10] also showed that untypeable O4,12:d:- was most frequently found in 29.1% (50/172) of all isolates, followed by serovar Typhimurium (15.1%) in 2003–2005. Our previous study showed that *S. enterica *Typhimurium is the leading serovar in the isolates from diarrheic pigs in 1996–2001[18]. Thus, *S. enterica *Typhimurium is likely to be predominant in *Salmonella *isolates from pigs in Japan.

The antimicrobial resistances patterns of the isolates are shown in Table 2. Higher resistance rates were observed against oxytetracycline (82.0%), dihydrostreptomycin (77.9%), kanamycin (41.0%) and trimethoprim (35.2%). Resistance rates to ampicillin, ceftiofur, bicozamycin, chloramphenicol and nalidixic acid were <10%. The MICs of apramycin, gentamicin, colistin, enrofloxacin, ofloxacin, and sulfadimethoxine were distributed unimodally. Two *S. enterica *Senftenberg isolates with MIC values higher than the breakpoint concentration (6.25 μg/ml) for ceftiofur were obtained from a broiler. The CTX-M-2 β-lactamase gene was detected in the ceftiofur-resistant isolates. In Japan, cephalosporins are not approved for the disease treatment in poultry. To date, extended spectrum β-lactamase (ESBL)-producing *E. coli *harboring the CTX-M-2 or CTX-M-18 β-lactamase has been obtained from broilers [7] and cattle in Japan [19]. ESBL-producing *S. enterica *Senftenberg obtained from river water was reported in Japan[20]. The β-lactamase gene type in these isolates was CTX-M-3 [20]. In addition, *S. enterica *Infantis strains resistant to cephalosporin were isolated from retail meats of domestic poultry in 2001–2003 [21] and in 2004–2005 [22]. Taguchi et al. [22] demonstrated that the cephalosporin-resistant *S. enterica *Infantis produced CMY-2 β-lactamase. The present study indicated that the CTX-M-2 β-lactamase producing *S. enterica *Senftenberg was prevalent in broiler chickens on the farm investigated before 1999. Thus, various types of β-lactamase producing *Salmonella *is found in the broiler chickens and the retail chicken meats in spite of non-approval for usage of cephalosporin antibiotics in chickens under the Japanese Pharmaceutical Affairs Law. We must continue to monitor the prevalence of cephalosporin-resistant *Salmonella *and should clarify the reasons why the resistant *Salmonella *has been prevalent in broiler industries.

**Table 2:** Antimicrobial susceptibility of *Salmonella *from food-producing animals

Antimicrobials	Resistant breakpoint (μg/mL)	MIC range (μg/mL)	MIC50 (μg/mL)	MIC90 (μg/mL)	No. of resistant isolates (%)							
					
					Broiler (n = 111)		Pigs (n = 10)		Cattle (n = 1)		Total (n = 122)	
Ampicillin	25	0.39->100	1.56	3.13	4	(3.6)	5	(50)	0	(0)	9	(7.4)
Ceftiofur	6.25	0.2–25	0.78	1.56	2	(1.8)	0	(0)	0	(0)	2	(1.6)
Apramycin		0.39–12.5	1.56	3.13								
Dihydrostreptomycin	100	6.25->100	>100	>100	88	(79.3)	6	(60)	1	(100)	95	(77.9)
Kanamycin	25	0.39->100	1.56	>100	49	(44.1)	0	(0)	1	(100)	50	(41.0)
Gentamicin		0.1–3.13	0.39	0.78								
Oxytetracycline	25	3.13->100	>100	>100	93	(83.8)	6	(60)	1	(100)	100	(82.0)
Bicozamycin	100	12.5->400	25	25	8	(7.2)	0	(0)	0	(0)	8	(6.6)
Chloramphenicol	50	0.78->100	3.13	6.25	0	(0)	4	(40)	0	(0)	4	(3.3)
Colistin		0.39–3.13	0.78	1.56								
Nalidixic acid	25	3.13->100	3.13	6.25	6	(5.4)	0	(0)	0	(0)	6	(4.9)
Enrofloxacin		≤0.05–0.39	≤0.05	0.1								
Ofloxacin		≤0.05–0.78	0.1	0.2								
Trimethoprim	3.13	0.1–6.25	0.39	6.25	42	(37.8)	1	(10)	0	(0)	43	(35.2)
Sulfadimethoxine		100->1600	>1600	>1600								

Most *S. enterica *Infantis isolates (91.3%) exhibited resistance to two or more of the tested antimicrobials (Table 3). In this study, *S. enterica *Infantis was the most frequently found serovar in broiler isolates obtained by first enrichment and DSEC. Resistance to dihydrostreptomycin, kanamycin, oxytetracycline and trimethoprim was found in 28.9% of the isolates by first enrichment, but in 61.3% by DSEC (Table 3). *S. enterica *Infantis isolates with similar resistance patterns were isolated using the two methods, although it is likely that there was a difference in the proportions of resistant isolates between the two methods.

**Table 3:** Antimicrobial resistance patterns of *S. enterica *Infantis by isolation methods


No. of antimicrobials	Antimicrobial resistance patterns ^a^	Broilers				Total (%)	
				
		FE^b^		DSEC^c^			

0	Susceptible	2	(5.3)	0	(0)	2	(2.9)

1	DSM	2	(5.3)	0	(0)	2	(2.9)
	OTC	2	(5.3)	0	(0)	2	(2.9)

2	DSM, OTC	9	(23.7)	8	(25.8)	17	(24.6)

3	DSM, KM, OTC	7	(18.4)	2	(6.5)	9	(13.0)
	DSM, OTC, TMP	4	(10.5)	2	(6.5)	6	(8.7)
	KM, OTC, TMP	1	(2.6)	0	(0)	1	(1.4)

4	DSM, KM, OTC, TMP	11	(28.9)	19	(61.3)	30	(43.5)

Total		38	(100)	31	(100)	69	(100)

^a ^DSM: dihydrostreptomycin, KM: kanamycin, OTC: oxytetracycline, TMP: trimethoprime

^b ^FE: First enrichment culture

^c ^DSEC: Delayed secondary enrichment culture

This study showed the prevalence of *Salmonella *in apparently healthy food-producing animals in Japan. In addition, CTX-M-2β-lactamase-producing *S. enterica *Senftenberg was isolated from broilers for the first time in 1999.

## Competing interests

The authors declare that they have no competing interests.

## Authors' contributions

KI provided data, discussed the results gained, and drafted. TT, AM, AK, KM, and TY provided data, discussed the results gained, and participated in revising the manuscript. TA discussed the results gained and revised the manuscript. All authors read and approved the final manuscript.
